# Do Diametric Measurements Provide Sufficient and Reliable Tumor Assessment? An Evaluation of Diametric, Areametric, and Volumetric Variability of Lung Lesion Measurements on Computerized Tomography Scans

**DOI:** 10.1155/2015/632943

**Published:** 2015-05-10

**Authors:** Aaron Frenette, Joshua Morrell, Kirk Bjella, Edward Fogarty, James Beal, Vijay Chaudhary

**Affiliations:** Sanford Health, 300 N. Seventh Street, Bismarck, ND 58501, USA

## Abstract

Diametric analysis is the standard approach utilized for tumor measurement on medical imaging. However, the availability of newer more sophisticated techniques may prove advantageous. An evaluation of diameter, area, and volume was performed on 64 different lung lesions by three trained users. These calculations were obtained using a free DICOM viewer and standardized measuring procedures. Measurement variability was then studied using relative standard deviation (RSD) and intraclass correlation. Volumetric measurements were shown to be more precise than diametric. With minimal RSD and variance between different users, volumetric analysis was demonstrated as a reliable measurement technique. Additionally, the diameters were used to calculate an estimated area and volume; thereafter the estimated area and volume were compared against the actual measured values. The results in this study showed independence of the estimated and actual values. Estimated area deviated an average of 43.5% from the actual measured, and volume deviated 88.03%. The range of this variance was widely scattered and without trend. These results suggest that diametric measurements cannot be reliably correlated to actual tumor size. Access to appropriate software capable of producing volume measurements has improved drastically and shows great potential in the clinical assessment of tumors. Its applicability merits further consideration.

## 1. Introduction

Measurement of tumor size is a critical factor in determining treatment options and monitoring treatment response. The American Joint Committee on Cancer staging system partially relies on the measured size of the primary tumor [1]. Choice of treatment and prognosis are then made based on the data received. Response to treatment is usually measured by reduction in the size of tumor or by necrosis. Two primary measures of tumor burden have been employed to determine prognosis and treatment: the World Health Organization (WHO) criteria and the Revised Response Evaluation Criteria in Solid Tumors (RECIST) [2, 3]. The WHO criteria utilize a maximum axial tumor diameter and its longest perpendicular diameter. The RECIST criteria rely on unidimensional measurement of the longest diameter (LD) [3].

Following treatment initiation, tumor response using revised RECIST criteria is then delineated into complete response (resolution of all known disease), partial response (reduction >30% of RECIST score), progressive disease (increase >20% of RECIST score), and stable disease (lack of partial response or progression).

**Figure 1:**
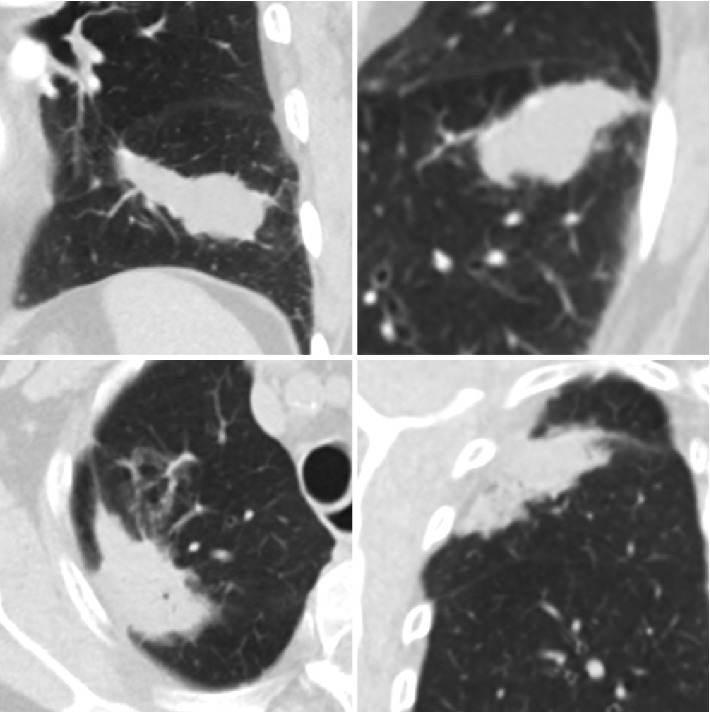
Depiction of abstract tumor morphology, four unique lung lesions.

Bearing in mind that treatment options and clinical decisions are based on these measurements, the importance of accuracy is vital. However, the objective and accurate measurement of tumors can be quite challenging.  Figure 1 highlights the primary challenge: tumors have abstract geometry. They vary in size, dimension, homogeneity, spiculation, density, and etcetera. These variances are not uncommon. With this forethought, the problem becomes apparent: can complex three-dimensional structures truly be assessed with traditional diametric techniques?

The purpose of this study was threefold. First, investigate the viability of volumetric measurement analysis, as compared to the standard diametric technique. Accordingly, the evaluation of measurement variability and practicality were paramount. The methods were designed to reflect that focus. A free and easily accessible software application was used to measure area and volume of solitary lung tumors, highlighting the ease and speed at which measurements can be taken. A relatively large sample size was used to compare interobserver variability amongst different measurement techniques. Second, determine whether diametric measurements can provide reliable tumor assessment. Third, propose future considerations to delineate measurement techniques, and improve clinical tumor assessment.

## 2. Materials and Methods

### 2.1. Patient Selection

Following Institutional Review Board approval from Sanford Medical Center, medical record numbers of all male and female patients who presented with radiologic evidence of lung cancer between 2000 and 2013 were obtained. The ICD-9 codes used in this study were 162.3 (malignant neoplasm of upper lobe bronchus or lung), 162.4 (malignant neoplasm of middle lobe bronchus or lung), and 162.5 (malignant neoplasm of lower lobe bronchus or lung). 

Of the patients surveyed, the first 24 patients with measurable lesions were identified. Lesions within the lung parenchyma were selected in a nondiscriminatory fashion; this allowed realistic size and morphology variation to be represented in the data. As such, the applicability of volumetric measurement analysis was tested over a wide range of conditions. With regard to the 64 unique lesions evaluated with volumetric measurements, the average size was 5.37 cm^3^, with a range of 0.19 cm^3^ to 63.89 cm^3^. Following anonymization of patient data, computerized tomography (CT) scans of these patients were then obtained from PACS and downloaded onto secured personal workstations.

### 2.2. Software Utilization

The DICOM viewer utilized for this study was OsiriX 32-bit open source version [4]. Additionally, there is a version that is FDA approved as a Class II Medical Device and certified as a European CE Class IIa for diagnostic imaging in medicine, which is available for a nominal price.

### 2.3. Procedure Standardization

For this study, three evaluators, Aaron Frenette, Joshua Morrell, and Kirk Bjella, were trained by a licensed radiologist with the goal of standardizing program usage and tumor border identification. During this training session, each user measured the diameter, area, and volume of the lesions under supervision to ensure similar measurement criteria were being met. It was agreed that while making measurements all evaluators would utilize the standard pulmonary window/level and proceed unrushed with attention to detail. These measurement standards were kept consistent throughout the entire study for all methods performed.

**Figure 2:**
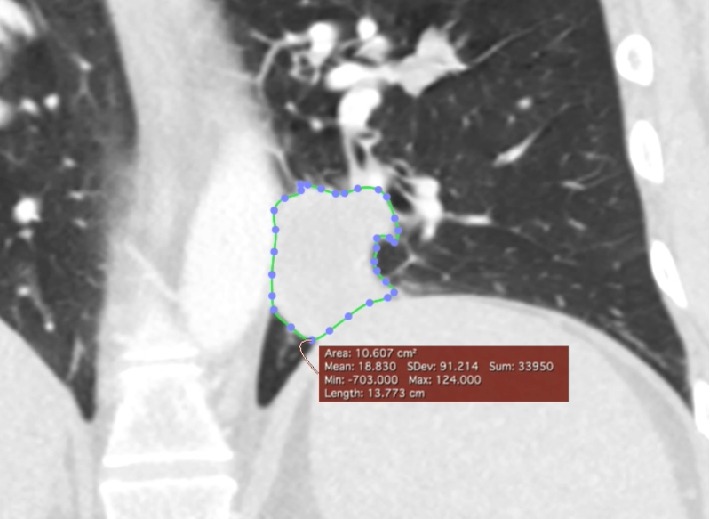
Depiction of a perimetric selection used to calculate the volume of a left lower lobe pulmonary nodule.

Following the training, each reader independently measured 64 unique lesions on 52 different CT scans using 3 mm slices with either axial or coronal section from 24 patients. Diameter, area, and volume measurement for each lesion were obtained. Diameter measurements were obtained by subjectively identifying the cross section with the longest diameter, followed by measuring the perceived longest diameter within that cross section using the “length” tool. The same cross section was used to calculate the area by outlining the perimeter of the lesion with the “closed polygon” tool. To calculate the volume of the lesion, the area was calculated for each cross section in which the tumor was present (Figure 2). Upon completion, “compute volume…” under the ROI menu was selected to calculate the lesion's volume.

### 2.4. Statistical Analysis

Measurements were recorded, combined, and analyzed in a spreadsheet (Microsoft Excel: Mac 2011 V14.1.0). The relative standard deviation (RSD) was calculated independently for diameter, area, and volume for each unique lesion. The RSD data was then averaged independently for diameter, area, and volume.

For each independent lesion, the diametric data was used to create an estimate of actual tumor size. Both area and volume were estimated and modeled as a circle and sphere, respectively. The appropriate mathematical relationships were applied: *A* = *π*∗(0.5∗*d*)^2^ and *V* = (4/3)∗*π*∗(0.5∗*d*)^3^. The approximated tumor size was then compared to the true tumor size, per volumetric measurements. RSD analysis was again applied to the estimated areas and volumes.

Analysis of variance (ANOVA) testing was applied to compare estimated area/volume to known measured area/volume. Additional data analysis was performed using SPSS, version 21 (SPSS Inc., Chicago, IL, USA). This software was used to quantify interobserver agreement and data reproducibility using intraclass correlation coefficient with 95% confidence intervals [5].

An internal evaluation of the software's volume calculation was carried out. This was performed by making a ballistics gel mold and suspending four clay objects. Their sizes and shapes were kept comparable to the study's lung lesions. A CT scan with 3 mm slices of the mold was obtained. Volumetric analysis was performed via OsiriX [6]. To determine the exact volume, the clay objects were again measured individually using water displacement. A small container was overfilled with water, allowing drainage via a runoff spout. The water level was allowed to equilibrate, so that any additional volume would cause drainage. The clay molds were carefully submerged. Water drained equal to the volume of the submerged object and was collected. The drainage was weighed to the nearest one-hundredth gram. The weight was then converted to volume, assuming water density of 1 g/mL. Finally, the software-measured volumes were compared against water displacement volumes.

## 3. Results

In the 64 independent CT imaged tumors, the RSD was lower in the areametric and volumetric measurements (3.95% and 4.04%, resp.), as compared to diametric measurements (4.65%). Diametric, areametric, and volumetric measurements all allowed for similarly low RSD. Estimated tumor dimensions, as compared to actual measured dimensions, showed considerably greater standard deviation of 9.31% for area and 13.95% for volume (Table 1).

**Figure 3:**
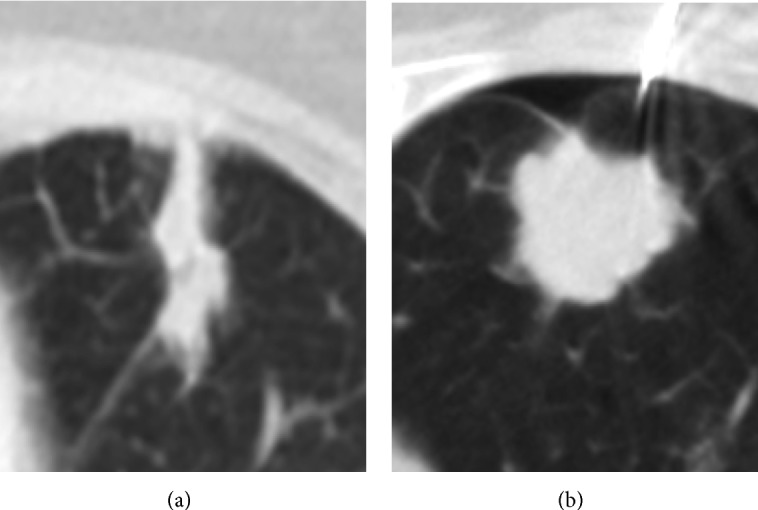
Depiction of poor diameter to size correlation.

**Figure 4:**
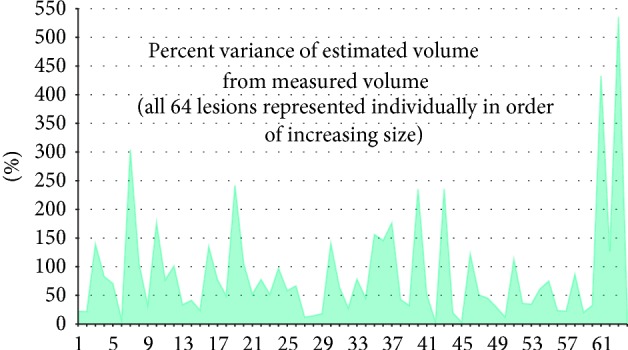


The estimated tumor dimensions demonstrated substantial variance from actual measured dimensions. On average, there was an 88.03% variance between the estimated and actual measured volumes. Variance was independent of tumor size and the range of this data was widely scattered, meaning no trend exists within the variance. (Table 2, Figure 4). These results suggest that diametric measurements cannot be correlated to actual tumor size.  Figure 3 represents one such example from the data.  Figure 3(a) has a diameter of 3.73 cm and a volume of 5.14 cm^3^.  Figure 3(b) has a diameter of 3.25 cm and a volume of 14.56 cm^3^. While these two independent lesions have similar diameters (Figure 3(a) > Figure 3(b) by 15%), their volumes diverge greatly (Figure 3(b) > Figure 3(a) by 183%). Image A also emphasizes the difficult task of consistent and objective placement of the diameter measurement.

Intraclass correlation coefficients (ICC) were calculated to assess interobserver reliability and reproducibility of the study. Different measurement techniques represent the “classes” in this study. The ICC calculates variance between different judges and compares that variance to total variance. ICC results were interpreted according to the following criteria: 0.00–0.20 slight reproducibility, 0.21–0.40 fair reproducibility, 0.41–0.60 moderate reproducibility, 0.61–0.80 substantial reproducibility, and >0.80 almost perfect reproducibility [7]. When scrutinizing the measurements of three different judges across 64 independent CT imaged tumors, the ICC verified minimal variance during the study. This was true for diametric, areametric, and volumetric measurements (Table 3).

The internal evaluation of the software's volume calculations using four clay test objects yielded an average deviation of 2.7% with a range of 0.297–6.692%. This mean deviation was less than that of the interobserver variability, which was 4.04%, indicating that the software is acceptably accurate at calculating true tumor volumes for this study.

## 4. Discussion

During the revision RECIST guidelines in 2009, volumetric analysis was not recommended, citing “lack of sufficient standardization and widespread availability” [3]. Our results argue otherwise. As used in this study, computer software capable of efficiently performing volumetric analysis is both economical and readily available. Diametric, areametric, and volumetric measurements were all shown to be reproducible, with high intraclass correlation coefficients and low relative standard deviation (Tables 3 and 1). Volumetric analysis did slightly outperform the other two techniques. This outcome alone does not necessarily support superiority but rather establishes viability. Vetted against conventional diametric methods, volumetric analysis was confirmed as a reliable measurement technique; standardization is undoubtedly achievable.

Diametric measurements lack accuracy and reliability in assessment of tumor size. Variance was both substantial (88.03% average) and unpredictable (3.10%–535.40% range, no association with tumor size). RSD values were also notably larger. The obvious opposing argument to this data is that tumors are not well represented as spheres, so why use spherical estimation? While the relationship may be imperfect, it was deemed acceptable for several reasons. The leap from tumor diameter to size occurs frequently in clinical medicine (e.g., RECIST criteria). To study this association, some type of objective scaling must be incorporated. Though a sphere is a reasonable choice, it must be understood that any model will create some degree of variance (inversely proportional to its accuracy). Hence, the trend in variance becomes arguably more important than average variance. If tumor diameter is truly statistically correlated with size, a variance trend will exist regardless of the chosen geometric model. Our results clearly demonstrate the absence of said trend. This finding is quite disconcerting with regard to its clinical implications. Simple diametric measurements are very limited in utility yet used almost exclusively. While the information they provide is minimal, the measurements are used for tumor assessment, which directly impacts clinical decisions. If diameter is statistically independent of tumor size, is our reliance on its use acceptable?

RECIST criteria are currently considered as the method of choice in assessing the response of a tumor to treatment. However, studies have shown discrepancies following review of investigators with secondary panels [8]. This discordance has been shown to be due to manual measurement variability [9, 10]. In some studies the measurement inaccuracies were able to account for 45% of misclassifications of tumor response [11]. In head to head studies between the two currently used methods, the RECIST and WHO criteria, discrepancies have ranged between 18 and 47% [8]. This variability was due to differences in the location, size, and shape of the tumors.

As shown, additional discrepancies exist between the measurement techniques themselves, which may worsen the inaccuracies of criteria classification. This reasoning is supported by other research, with one study concluding only “fair to poor agreement in treatment response classification” among different measurement techniques [12].

Volumetric analysis of growth rates has been reported to be helpful in differentiating between benign and malignant lesions [13]. Some small population studies have demonstrated an advantage with automated volumetric analysis [8]. Many studies have shown a reduction of interobserver variability with the use of computer-assisted volume measurements [14]. Importantly, computer-assisted measurements allow for more reproducible results when compared to other measurement techniques [13, 15]. It has been shown that volume measurements may allow earlier recognition of tumor response to treatment and may predict clinical response earlier than RECIST [16, 17]. However, most of these studies used small sample sizes.

As shown by the results of our internal evaluation using clay models, CT volumetric analysis is very accurate. This result is shared by other studies, including those focused on very small nodules. One study looked specifically at nodules measuring less than 10 mm in diameter and found that the volume could be measured accurately to within ±3% [18]. Another study showed that volumetric analysis could characterize small tumors (5–10 mm) dramatically better than the RECIST criteria. The study detailed the necessity for volumetric analysis when evaluating changes in lesions of this size [19].

There were limitations to this study. First, the retrospective design of this study predisposes it to miscodification bias as well as selection bias. Second, the study was lacking a validation cohort. Third, lesions were limited to lung parenchyma to allow consistency throughout the study. Fourth, 3 mm slices were used in this study, rather than the 1.5–2 mm slices utilized in many follow-up studies of pulmonary nodules. The 3 mm slices would naturally underestimate overall tumor volume when compared to thinner slices. Fifth, volumetric measurements may be susceptible to some error that similarly affects diametric measurements (e.g., change of tumor size during respiration for pulmonary lesions and change in tumor enhancement during different contrast phases). Sixth, performing volumetric analysis is more labor intensive than diametric analysis. Under repeated trials, a study lesion of average size (5.37 cm^3^) could be consistently measured in less than two minutes. Time trials were conducted with adherence to the same technical standards used for data collection: unrushed with attention to detail. A two-minute measurement time is reasonably low for an average lesion, but added factors may exacerbate this time (e.g., larger lesions, multiple lesions, and smaller slices). Labor time is perhaps the greatest limiting factor toward incorporating volumetric measurement analysis in practical medicine. Yet, this limitation is certainly not impassable. Tools that aid in speeding up this process are already being developed (e.g., abovementioned automated methods). Continued research may offer a better understanding of this technique and more efficient ways to use it. Seventh, the readers of this study were senior medical students with limited radiologic training. Experienced radiologists may be able to perform measurements with even greater efficiency and speed. Ideally, a larger number of trained radiologists would be used in this type study and metastases in many different organ systems would be assessed, including tumors presenting in areas with surrounding tissue of similar density.

Future investigation is warranted. The utility of volumetric analysis likely is not limited to lung parenchyma lesions. Rather, the technique possesses potential for wide applicability in medical science (e.g., evaluation of other anatomic structures). It would be useful to again compare different measurement techniques in patients receiving chemotherapy, evaluating tumor evolution over time. Judging by the aforementioned inconsistencies, it is unlikely that tumor diameter will change proportionally with overall tumor size. Volumetric analysis may be able to detect tumor response sooner, thereby significantly improving patient outcomes. It has already been shown that alternative methods such as PET scan analysis can save patients from receiving unnecessary doses of chemotherapy [20]. Volumetric analysis is able to deliver a much more complete picture of overall tumor size. It can provide oncologists with additional and more accurate information to tailor treatment regiments, better equipping them to treat disease whilst mitigating side effects.

## 5. Conclusion

This study emphasizes that volumetric measurements can be accomplished on readily accessible software with highly reproducible results. It also stresses the limitations of diametric measurements. An industry standard, diametric analysis allows the clinical radiologist to make fast and reproducible measurements. Unfortunately, this method offers little insight into the true size of a tumor. Unsurprisingly, RECIST studies also document other discrepancies. As imaging technology advances, so must our associated practices. The utilization of a volumetric method provides a more comprehensive and accurate assessment of tumor size, which may alter clinical decisions. The method boasts promising potential and may have widespread applicability. Its use deserves further consideration.

## Figures and Tables

**Table 1:** Interobserver variability among measurements: relative standard deviation.

Diametric	4.65%
Areametric	3.95%
Areametric, estimated	9.31%
Volumetric	4.04%
Volumetric, estimated	13.95%

**Table 2:** Average percent variance of estimated volume from measured volume.

	Variance	Range	Chi^2^	*p*
Areametric	43.50%	4.74%–221.76%	0.31	0.58
Volumetric	88.03%	3.10%–535.40%	0	1

**Table 3:** Analysis of interobserver reliability/reproducibility: intraclass correlation.

	Intraclass correlation	95% CI lower bound	95% CI upper bound
Diametric, single	.979	.969	.987
Diametric, average	.993	.989	.996
Areametric, single	.998	.997	.999
Areametric, average	.999	.999	1.000
Volumetric, single	.999	.999	1.000
Volumetric, average	1.000	1.000	1.000
